# Poly(A) RT–PCR measurement of diagnostic genes in pancreatic juice in pancreatic cancer

**DOI:** 10.1038/sj.bjc.6606047

**Published:** 2011-01-18

**Authors:** M Oliveira-Cunha, R J Byers, A K Siriwardena

**Affiliations:** 1 Hepatobiliary Surgery Unit, Manchester Royal Infirmary, Oxford Road, Manchester M13 9WL, UK; 2 Histopathology Department, Manchester Royal Infirmary, Manchester, UK

**Keywords:** poly(A) cDNA, RT–PCR, quantitative RT–PCR, pancreatic cancer and gene expression in pancreatic cancer

## Abstract

**Background:**

The last decade has seen significant progress in understanding the molecular biology of pancreatic adenocarcinoma. There is now an urgent need to translate these molecular techniques to clinical practice in order to improve diagnosis and prediction of response to treatment. The objectives of this study are to utilise poly(A) RT–PCR to measure expression levels of diagnostic *Indicator* genes, selected from microarray studies, of RNA from intraoperatively sampled pancreatic ductal juice and to correlate these expression levels with those in matched pancreatic tissue resection samples.

**Methods:**

Intraoperative sampling of pancreatic juice and collection of matched tissue samples was undertaken in patients undergoing pancreaticoduodenectomy for suspected tumour. RNA was isolated and poly(A) PCR and real-time PCR used to measure expression levels of 30 genes. Spearman's rank correlation test was used to examine the relationship of gene expression between pancreatic juice and tissue.

**Results:**

Of the 30 *Indicator* genes measured, just one, *ANXA1*, showed a significant correlation of expression level between pancreatic juice and tissue samples, whereas three genes, *IGFBP3* (*P*⩽0.035), *PSCA* (*P*⩽0.001) and *SPINK1* (*P*⩽0.05), showed significantly different expression between cancerous and benign pancreatic tissue samples.

**Conclusions:**

These results demonstrate that RNA analysis of pancreatic juice is feasible using the poly(A) cDNA technique, that correlation of gene expression exists between pancreatic juice and tissue for very few genes and that gene expression profiling can distinguish between benign and malignant pancreatic tissue. This indicates possible use of the technique for measurement of *Indicator* genes in pancreatic tissue for diagnosis of pancreatic cancer from very small tissue samples.

Carcinoma of the pancreas (PC) is an aggressive disease and the fourth leading cause of cancer-related mortality in the Western world (Jemal *et al*, 2009). The 5-year survival rates for affected patients are a dismal 3%, emphasising the need for studies to better understand the molecular biology of pancreatic cancer and to translate this knowledge to clinical practice (Neoptolemos *et al*, 2001).

Increased knowledge of the molecular basis of cancer in general has led to a better understanding of the pathobiology of most cancers, with the possibility of improved diagnosis and development of novel treatments; this is also the case for pancreatic cancer. Recent advances in global gene expression profiling have provided molecular markers for cancer diagnosis, and there is a need to develop methods for their measurement in routine clinical samples.

Global cDNA amplification, by poly(A) PCR, allows marker genes to be detected at very low levels, improving sensitivity, while also providing an improved level of specificity through the ability to analyse multiple genes (Brady and Iscove, 1993; Sakhinia *et al*, 2005; Byers *et al*, 2008). Critically, global cDNA permits analysis of very small numbers of cells. This is of particular relevance in enabling measurement of gene expression profiles from cells isolated from pancreatic juice, providing a relatively noninvasive method for diagnosis (Oliveira-Cunha *et al*, 2010a).

Additionally, the poly(A) PCR method can produce an essentially permanent archive of representative cDNA suitable for screening with multiple gene-specific probes. Furthermore, the method is rapid (all samples can be prepared and screened within 48 h) and of particular importance for clinical use (Brady *et al*, 1995; Sakhinia *et al*, 2005; Byers *et al*, 2008; Oliveira-Cunha *et al*, 2010a, 2010b).

This study explores the feasibility of gene expression profiling from RNA isolated from pancreatic ductal juice and tests the hypothesis that detection of gene signatures, the so-called *Indicator* genes, measured by globally amplified poly(A) cDNA in ductal juice from patients with pancreatic cancer will provide a high degree of correlation with *Indicator* genes detected by the same method in the tumour samples of the same patients. To test this hypothesis, RNA was extracted from pancreatic juice and tissue obtained intraoperatively from patients undergoing pancreaticoduodenectomy. Poly(A) PCR was used to globally amplify the RNA followed by measurement of the expression levels of *Indicator* genes by real-time PCR. The gene expression profiles for pancreatic juice were then compared with those for the parallel pancreatic tissue samples.

## Materials and methods

### Study population

Pancreatic juice was collected by intraoperative aspiration of the main pancreatic duct in patients undergoing pancreaticoduodenectomy at Manchester Royal Infirmary between May 2007 and June 2009. For all patients a matched tissue sample from the main tumour was obtained from the fresh specimen under direct vision in the histopathology laboratory. The study protocol had local ethical committee approval and written informed consent was obtained from all patients. In all, 45 patients were included; sufficient pancreatic juice was obtained in 41 patients with matched pancreatic tissue/pancreatic juice in 34 of these patients.

Histological analysis confirmed pancreatic/periampullary malignancy in 37 cases (11 ductal adenocarcinomas, 14 ampullary carcinomas, 3 cholangiocarcinomas, 4 intraductal papillary mucinous neoplasms (IPMNs), 2 neuroendocrine tumour, 2 mucinous cystic neoplasms (MCNs) and 1 acinar cell carcinoma). Of the non-cancer group, seven were chronic pancreatitis and one autoimmune pancreatitis. In the subset of 34 patients with matched pancreatic tissue and juice, the diagnoses were: 10 ductal adenocarcinomas, 12 ampullary carcinomas, 2 cholangiocarcinomas, 1 neuroendocrine tumour, 3 IPMNs, 1 MCN, 1 autoimmune pancreatitis and 4 chronic pancreatitis.

### Extraction of RNA and global amplification of polyadenylated mRNAs (poly(A) RT–PCR)

Isogen-LS (Wako, Osaka, Japan) was used in combination with RNeasy mini Kit (Qiagen, Crawley, UK) for total RNA extraction and purification from pancreatic juice. Total RNA from pancreatic tissue was extracted following the RNeasy kit (Qiagen, Hilden, Germany) according to the manufacturer's instructions. Samples were then quantified using a NanoDrop ND-1000 spectrophotometer (NanoDrop Technologies, Wilmington, DE, USA) analysis and RNA integrity was checked by Agilent 2100 Bioanalyzer (Agilent Technologies, Waldbronn, Germany) according to the manufacturer's instructions. The poly(A) PCR method has three steps, namely poly(A) primed reverse transcription to produce a first-strand cDNA for each mRNA; polyadenylation of the first-strand cDNA, rendering it defined at both ends; and polymerase chain reaction using a poly(dT) primer, which anneals to the poly(A) site on the first-strand cDNA. We performed poly(A) PCR on RNA isolated following standard published protocols.

### TaqMan real-time quantitative PCR

TaqMan PCR primers and probes were designed for 30 *Indicator* genes that have previously been reported as overexpressed in pancreatic cancer together with two housekeeping genes (*GAPDH* and *PSMB6*) using Custom TaqMan Gene Expression Assays (Applied Biosystems, Foster City, CA, USA); details of all primers and probes used are listed in Supplementary Table 1 online. The genes studied were selected after literature review of microarray data. The selected genes include a range of genes covering lineage markers, cell cycle markers and activation markers in order to maximise possibility of detection of genes that are overexpressed in pancreatic cancer, metastatic disease, ductal epithelial cells and chronic pancreatitis. All PCR primer pairs were designed for an mRNA sequence within 300 base pair (bp) of the poly(A) signal of each *Indicator* gene.

The real-time PCR reactions were performed in 96-well optical reaction plates, in a final volume of 25 *μ*l, containing 12.5 *μ*l of TaqMan Universal PCR Mastermix (Applied Biosystems), 1.25 *μ*l of Custom TaqMan Gene Expression Assays Primers and probes (Applied Biosystems), 8.75 *μ*l of UltraPure DEPC water (Invitrogen, Carlsbad, CA, USA) and 2.5 *μ*l of cDNA. All samples were analysed using an ABI Prism 7900HT sequence detection system (Applied Biosystems).

Amplification plots indicating fluorescence intensity at each cycle were obtained from which Ct values were measured for each sample. PCRs were run in triplicates for each sample and Ct averages were obtained. The average Ct value for each gene for each patient was calculated from the triplicates, followed by normalisation to the average of the two housekeeping genes following the 2^ΔΔCt^ method (Livak and Schmittgen, 2001; Schefe *et al*, 2006; Yuan *et al*, 2008).

### Statistical analysis

Statistical analyses were carried out using Excel (Microsoft, Redmond, WA, USA) and SPSS (Statistical Package for Social Sciences, Woking, Surrey, UK) version 17. Relative fold changes were calculated using the ΔΔCt method (Livak and Schmittgen, 2001; Schefe *et al*, 2006; Yuan *et al*, 2008).

The data were not normally distributed; therefore, nonparametric tests were used. Spearman's rank correlation was used to examine the relationship between pancreatic juice and pancreatic tissue samples. Statistical analysis of the expression levels of the 30 *Indicator* genes in the benign and cancer groups was performed using the Mann–Whitney test, with a *P-*value of at least 0.05 for statistical significance. As the expression levels for all the genes fall across a wide range, the log_10_ scale was used to plot all values to aid visual comparison. The ranking of the expression levels of *Indicator* genes between samples was presented as the mean rank statistical difference. False discovery rate analysis was used to control for multiple comparisons. The *q*-value is defined to be the FDR analogue of the *P*-value and should be <0.05 to be considered significant.

## Results

### RNA extraction

Total RNA was successfully extracted from all 41 samples of pancreatic juice and all 34 samples of pancreatic tissue.

For pancreatic juice the median RNA yield was 68.45 ng *μ*l^–1^ (range from 2.75 to 1418 ng *μ*l^–1^, s.d.±268.3 ng *μ*l^–1^). RNA quality, assessed by RNA integrity number (RIN), gave a median RIN of 5.5 (range from 1.9 to 10). Analysis of the RIN, purity ratios and gel electrophoretic results in combination demonstrated good-quality RNA in 25 cases and poor-quality RNA in 16 cases.

For pancreatic tissue, the median RNA yield was 132 ng *μ*l^–1^ (range from 3.8 to 2594 ng *μ*l^–1^, s.d.±643 ng *μ*l^–1^). The median purity ratio 260/280 was 2.09 (range from 1.41 to 2.15, s.d.±0.19) for total RNA of pancreatic tissue.

### Poly(A) PCR

Poly(A) cDNA was generated from mRNA extracted from all samples of pancreatic juice and tissue. The cDNA quantity, assessed by gel electrophoresis, generated from each sample, was highly reproducible over at least duplicate tests, demonstrating the reliability of the method.

### Real-time PCRs

**Figure 1:**
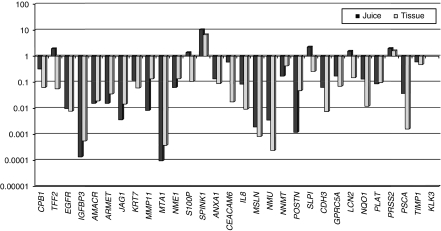
Gene expression in 30 *Indicator* genes after normalisation to reference genes. This graph illustrates the median gene expression values (Ct values in log scale, after normalisation for *GAPDH* and *PSMB6*) in pancreatic juice and tissue samples. Pancreatic juice and tissue were tested for 30 *Indicator* genes. The *KLK3* gene was not expressed in any of the samples tested.

After real-time PCR, Ct values were obtained for each sample and gene. Samples were normalised according to Ct values of the average for both housekeeping gene in each sample. Figure 1 illustrates the median gene expression levels (median ΔCt values in logarithmic scale) of all genes tested.

### Gene expression levels in pancreatic juice compared with pancreatic tissue samples

Median gene expression levels in pancreatic juice were similar to that in the corresponding resection specimen, with the exception of *TFF2* and *S100P*. A statistically significant correlation was present for *ANXA1* (*P*⩽0.043), *MSLN* (*P*⩽0.037) and *PLAT* (*P*⩽0.046). However, these *P-*values are results of multiple comparisons; therefore, false discovery rate (FDR) control was used for correction. The correlation coefficients are shown in Table 1; none of the 30 genes were significant by FDR at a *q*-value of <0.05.

As tumoral cells are shed into the pancreatic duct and juice in ductal adenocarcinoma, the number of malignant cells in pancreatic juice may be higher in these cases. For this reason, subgroup analysis of 30 *Indicator* genes using Spearman's correlation test was performed in the ductal adenocarcinoma samples (*n*=11). Expression levels between pancreatic juice and tissue samples in *ANXA1* gene were correlated with *q*-values <0.05, Q⩽0.002; the correlation coefficients are shown in Table 2.

### Gene expression levels in pancreatic juice and tissue samples in pancreatic cancer compared with chronic pancreatitis

**Figure 2:**
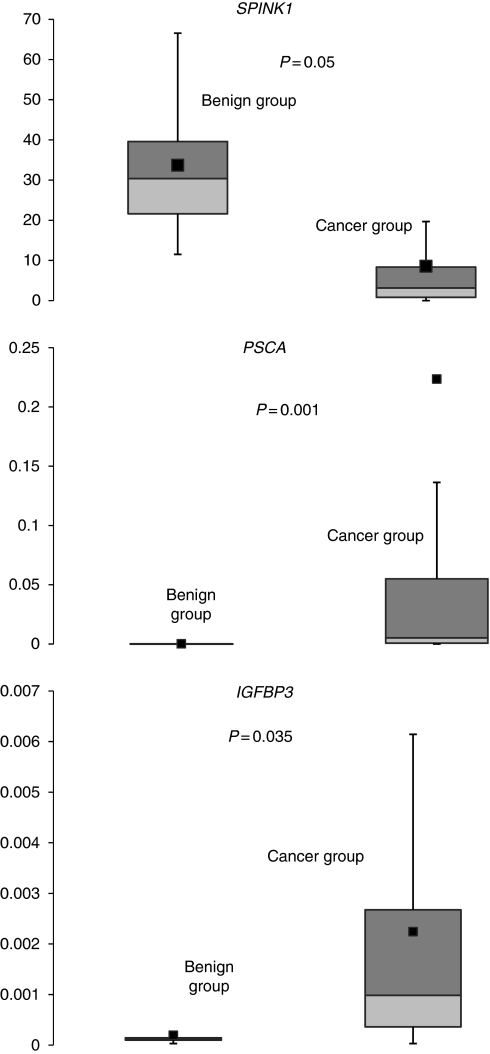
Expression levels of *SPINK1*, *PSCA* and *IGFBP3* in pancreatic tissue with statistically significant difference between patients with either benign or malignant disease. *SPINK 1*, *PSCA* and *IGFBP3* were statistically significantly higher in the benign group compared with cancerous group; for each, maximum and minimum values are shown, together with 25, 50 and 75% confidence intervals and the means (dark squares).

Mann–Whitney *U-*test did not identify any genes with statistically different expression between cancer and benign samples in pancreatic juice samples. Of the 30 *Indicator* genes studied in pancreatic tissue, only *KLK3* was not expressed in pancreatic cancer or chronic pancreatitis samples. Mann–Whitney *U* test identified three genes, namely *IGFBP3* (*P*⩽0.035), *PSCA* (*P*⩽0.001) and *SPINK1* (*P*⩽0.055), with statistically different expression between cancerous and benign pancreatic tissue samples (Figure 2).

## Discussion

Use of gene expression profiles represents an innovative approach to cancer classification and prognostication and has been applied to an increasingly wide range of cancers. Measurement of these gene signatures by real-time PCR would enable translation to clinical use, whereas their analysis in pancreatic juice would allow noninvasive diagnosis and monitoring. This study tests whether these gene signatures can be identified in pancreatic ductal juice as this is a relatively easily accessible biological material. For the purposes of this study, we have compared gene signatures in ductal juice with those in the pancreatic tumour specimen in the same patient. As not all patients undergoing resection had adenocarcinoma, we have also examined for differences in gene expression profile between juice and pancreatic tissue in patients with either benign disease or cancer.

This study demonstrated that three genes, *IGFBP3*, *SPINK1* and *PSCA*, can differentiate, in tissue samples, cancerous samples from benign samples (normal and chronic pancreatitis cases). It also aimed to identify, from candidate *Indicator* genes in the literature, genes predictive of pancreatic cancer in pancreatic juice. There was a significant correlation between *ANXA1* gene expressed in pancreatic cancer juice and tissue samples when ductal adenocarcinoma samples were analysed as a discrete cohort. Annexin I belongs to a family of Ca^2+^-dependent phospholipid-binding proteins with phospholipase A2 inhibitory activity. Annexin I is a steroid-regulated protein and thus implicated in some actions of glucocorticoids, including inhibition of cell proliferation, anti-inflammatory effects, the regulation of cell migration, differentiation, death and the hypothalamic–pituitary axis. The molecular mechanisms and the clinical significance of annexin I altered expression in malignancies still remain a debate. Results from several studies demonstrated that overexpression of *ANXAI* is a frequent event in pancreatic cancer, which may be one of the factors associated with malignant transformation, de-differentiation and poor prognosis of pancreatic cancer (Han *et al*, 2002; Iacobuzio-Donahue *et al*, 2002; Iacobuzio-Donahue *et al*, 2003a, 2003b; Bai *et al*, 2004; Grutzmann *et al*, 2004).

The lack of significant statistical correlation between gene expression in pancreatic juice and tissue samples for the majority of the *Indicator* genes may be because of the small amount of RNA in pancreatic juice and to RNA degradation by RNases, which could affect certain transcripts more than others. Additionally, there is no clear evidence as to whether the origin of the differentially expressed mRNAs in the pancreatic juice is from secreted RNA or released tumour cells, and the different mRNA levels found in the pancreatic juice compared with the solid tumour may merely reflect a sub-population of mRNAs that are preferentially secreted or cells that are preferentially shed that are nevertheless indicative of pancreatic cancer (Tomlinson and Korc, 2006). In addition, the mRNAs measured in pancreatic juice may reflect cells involved in inflammatory responses.

Three genes, *IGFBP3*, *SPINK1* and *PSCA*, showed differential expression between cancerous and benign samples. The *IGFBP3* is a gene member of the insulin-like growth factor binding protein (IGFBP) family, and encodes a protein with an IGFBP domain and a thyroglobulin type-I domain (Hansel *et al*, 2004). Insulin-like growth factors (IGFs) are multifunctional peptides that regulate cell proliferation, differentiation and apoptosis elements important in carcinogenesis. IGFBP-3 is the main binding protein of IGF-I, and it circulates in the plasma, prolonging the half-life of IGFs and altering their interaction with cell surface receptors (Hansel *et al*, 2004; Renehan *et al*, 2004). In agreement with previous microarray studies, the present study has demonstrated that *IGFBP3* is overexpressed in pancreatic cancer and can differentiate cancerous from benign tissue (Iacobuzio-Donahue *et al*, 2002; Logsdon *et al*, 2003; Iacobuzio-Donahue *et al*, 2003a; Grutzmann *et al*, 2004; Missiaglia *et al*, 2004; Laurell *et al*, 2006).

The *SPINK1* gene encodes pancreatic secretory trypsin inhibitor (PSTI) and tumour-associated trypsin inhibitor (TATI), which is secreted from ductal cells into pancreatic juice. PSTI is thought to function in the prevention of trypsin-catalysed premature activation of zymogens within the pancreas and the pancreatic duct. The serum levels of PSTI increase in association with severe inflammatory association, tissue damage and major surgery, all of which suggest that PSTI has a role in the acute-phase reaction (Graf and Bimmler, 2006; Paju and Stenman, 2006). The *SPINK1* gene is normally not expressed outside of the pancreas, but its expression has been demonstrated to be elevated in pancreatic cancer (Friess *et al*, 2001; Iacobuzio-Donahue *et al*, 2002; Iacobuzio-Donahue *et al*, 2003a; Grutzmann *et al*, 2004; Fukushima *et al*, 2005), whereas the present study demonstrated that *SPINK1* is highly expressed in normal pancreatic tissue compared with pancreatic cancer.

The *PSCA* gene encodes a glycoprotein that is anchored to the cell membrane. Real-time PCR and immunohistochemistry studies have demonstrated that *PSCA* has a limited normal tissue distribution and is most strongly expressed in cells of the prostate (Reiter *et al*, 1998). Recently, immunohistochemistry and northern blot studies revealed that normal pancreatic tissue does not express *PSCA*, with the exception of a single case of atrophic pancreatic ducts (Reiter *et al*, 1998; Argani *et al*, 2001). This study has demonstrated that *PSCA* is overexpressed in pancreatic cancer and poorly expressed in normal pancreas and chronic pancreatitis, and similar results have been found in microarray and real-time studies (Argani *et al*, 2001; Ryu *et al*, 2002; Missiaglia *et al*, 2004; Grubbs *et al*, 2006).

RNA extraction and quality is a critical step in gene expression studies. To date, RNA extraction from pancreatic tissue and juice processing has not been standardised, leading to unanswered concerns of how to best store the collected specimen and maintain reproducibility. The development of effective and reproducible RNA isolation techniques depends on inhibition of contaminating RNases, enrichment of messenger RNA species and elimination of contaminating DNA (Kiba *et al*, 2007). In this study, to limit RNA degradation, pancreatic juice and tissue were placed immediately into an RNase inhibitor solution. To assure good RNA quality, four different methods of RNA extraction were tested and the method that generated the highest purity ratio (A260/A280 and A230/A260) by spectrophotometric analysis was chosen as the final method; RNA quality was assessed by RIN measurement and was good in a majority of samples. As variability in the expression of reference genes may lead to false results, this study considered all the evidence published in the literature and selected two reference genes, *PSMB6* (*proteasome subunit Y*) and *GAPDH* (*glyceraldehyde-3-phosphate dehydrogenase*), with which RNA concentrations were normalised in quantitative RT–PCR analyses (Rubie *et al*, 2005).

In summary, this study provides important, novel confirmatory evidence of the feasibility and utility of RNA analysis of pancreatic juice and tissue by global cDNA amplification using poly(A) PCR followed by real-time PCR measurement of specific genes. The poly(A) cDNA technique is a sensitive, reproducible, robust and nonexpensive technique for the analysis of RNA from pancreatic tissue. However, poor quality may limit its application. RNA integrity number should be part of an integral report in RNA studies. Of particular relevance to clinical practice is the utility to work with small index sample volumes and to generate results in a short time frame. The results indicate that *ANXA1* in pancreatic ductal juice has translational potential as a marker of pancreatic cancer. Furthermore, the differential expression patterns of *SPINK1*, *PSCA* and *IGFBP3* may be of value in the differentiation of pancreatic cancer from chronic pancreatitis.

## Figures and Tables

**Table 1:** Spearman's correlation coefficients of pancreatic juice *vs* pancreatic tissue

**Gene**	**Sig. (two-tailed) *P-*value**	***Q-*value (FDR)**	**Gene**	**Sig. (two-tailed) *P-*value**	***Q*-value (FDR)**
*CPB1*	0.328	0.791	*IL8*	0.355	0.791
*TFF2*	0.940	0.971	*MSLN*	0.037^*^	0.444
*EGFR*	0.946	0.971	*NMU*	0.229	0.791
*IGFBP3*	0.732	0.941	*NNMT*	0.639	0.941
*AMACR*	0.770	0.941	*POSTN*	0.528	0.941
*ARMET*	0.772	0.941	*SLPI*	0.712	0.941
*JAG1*	0.874	0.971	*CDH3*	0.209	0.791
*KRT7*	0.228	0.791	*GPRC5A*	0.560	0.941
*MMP11*	0.311	0.791	*LCN2*	0.176	0.791
*MTA1*	0.779	0.941	*NQO1*	0.639	0.941
*NME1*	0.283	0.791	*PLAT*	0.046^*^	0.444
*S100P*	0.479	0.926	*PRSS2*	0.311	0.791
*SPINK1*	0.885	0.971	*PSCA*	0.971	0.971
*ANXA1*	0.043^*^	0.444	*TIMP1*	0.105	0.761
*CEACAM6*	0.475	0.926	*KLK3*	Not expressed	Not expressed

Abbreviations: *ANXA1*=*annexin A1*; ARMET=now called MANF, mesencephalic astrocyte-derived neurotrophic; *AMACR*=*α-methylacyl-CoA racemase*; *CEACAM6*=*carcinoembryonic antigen-related cell adhesion molecule 6 (non-specific cross reacting antigen)*; *CDH3*=*cadherin 3, type 1, P-cadherin (placental)*; CPB1=carboxypeptidase B1; *EGFR*=*epidermal growth factor receptor*; FDR=false discovery rate; GPRC5A=G protein-coupled receptor family C, group5 member A factor; *IL8*=*interleukin 8*; *IGFBP3*=*insulin-like growth factor binding protein 3*; *JAG1*=*jagged 1*; *KLK3*=*kallikrein-related peptidase 3*; *KRT7*=*keratin 7*; *LCN2*=*lipocalin 2*; *MSLN*=*mesothelin*; *MMP11*=*matrix metallopeptidase 11 (stromelysin 3)*; *MTA1*=*metastasis associated 1*; *NQO1*=*NAD(P)H dehydrogenase, quinone 1*; *NMU*=*neuromedin U*; *NNMT*=*nicotinamide N-methyltransferase*; *NME1*=*non-metastatic cells 1, protein (NM23A) expressed in*; *POSTN*=*periostin, osteoblast specific factor*; *PLAT*=*plasminogen activator, tissue*; *PRSS2*=*protease, serine, 2 (trypsin 2)*; *PSCA*=*prostate stem cell antigen*; *S100P*=*S100 calcium binding protein P*; *SPINK1*=*serine peptidase inhibitor, Kazal type 1*; *SLPI*=*secretory leukocyte peptidase inhibitor*; *TFF2*=*trefoil factor 2*; *TIMP*=*TIMP metallopeptidase inhibitor 1*. ^*^P<0.05.

**Table 2:** Spearman's correlation coefficients of pancreatic juice *vs* pancreatic tissue in ductal adenocarcinoma samples only

**Gene**	**Sig. (two-tailed) *P-*value**	***Q*-value (FDR)**	**Gene**	**Sig. (two-tailed) *P-*value**	***Q*-value (FDR)**
*CPB1*	0.108	0.358	*IL8*	0.265	0.421
*TFF2*	0.770	0.446	*MSLN*	0.667	0.444
*EGFR*	0.019^*^	0.145	*NMU*	0.104	0.358
*IGFBP3*	0.667	0.444	*NNMT*	0.610	0.444
*AMACR*	0.200	0.421	*POSTN*	0.787	0.446
*ARMET*	0.787	0.446	*SLPI*	0.405	0.421
*JAG1*	0.397	0.421	*CDH3*	0.505	0.429
*KRT7*	0.787	0.446	*GPRC5A*	0.433	0.421
*MMP11*	0.289	0.421	*LCN2*	0.332	0.421
*MTA1*	0.624	0.444	*NQO1*	0.955	0.521
*NME1*	0.294	0.421	*PLAT*	0.420	0.421
*S100P*	0.987	0.521	*PRSS2*	0.117	0.358
*SPINK1*	0.326	0.421	*PSCA*	0.468	0.421
*ANXA1*	0.002^*^	0.03	*TIMP1*	0.603	0.444
*CEACAM6*	0.460	0.421	*KLK3*	Not expressed	Not expressed

Abbreviations: ARMET=now called MANF, mesencephalic astrocyte-derived neurotrophic; *AMACR*=*α-methylacyl-CoA racemase*; *ANXA1*=*annexin A1*; CPB1=carboxypeptidase B1; *CDH3*=*cadherin 3, type 1, P-cadherin (placental)*; *CEACAM6*=*carcinoembryonic antigen-related cell adhesion molecule 6 (non-specific cross reacting antigen)*; *EGFR*=*epidermal growth factor receptor*; FDR=false discovery rate; GPRC5A=G protein-coupled receptor family C, group5 member A factor; *IL8*=*interleukin 8*; *IGFBP3*=*insulin-like growth factor binding protein 3*; *JAG1*=*jagged 1*; *KLK3*=*kallikrein-related peptidase 3*; *KRT7*=*keratin 7*; *LCN2*=*lipocalin 2*; *MSLN*=*mesothelin*; *MMP11*=*matrix metallopeptidase 11 (stromelysin 3)*; *MTA1*=*metastasis associated 1*; *NQO1*=*NAD(P)H dehydrogenase, quinone 1*; *NMU*=*neuromedin U*; *NNMT*=*nicotinamide N-methyltransferase*; *NME1*=*non-metastatic cells 1, protein (NM23A) expressed in*; *PLAT*=*plasminogen activator, tissue*; *PRSS2*=*protease, serine, 2 (trypsin 2)*; *PSCA*=*prostate stem cell antigen*; *POSTN*=*periostin, osteoblast specific factor*; *S100P*=*S100 calcium binding protein P*; *SPINK1*=*serine peptidase inhibitor, Kazal type 1*; *SLPI*=*secretory leukocyte peptidase inhibitor*; *TFF2*=*trefoil factor 2*; *TIMP*=*TIMP metallopeptidase inhibitor 1*. ^*^P<0.05.
