# Supplementary material for: Poly(A) RT–PCR measurement of diagnostic genes in pancreatic juice in pancreatic cancer
Source: Br J Cancer. 2011 Jan 18;104(3):514–9. doi: 10.1038/sj.bjc.6606047 (PMC3049557; doi:10.1038/sj.bjc.6606047)
Supplement: Supplementary Table [file 6606047x1.doc]

**Supplementary Table**

Table 1 – Housekeeping and Indicator genes

| **Gene** | **Accession Number** | **Primers** |
| --- | --- | --- |
| AMACR | NM_014324 | TCAGTTTTAGGGTTGCCTGTATCC  GAAAGCTGACAGCCCAGAGA  CAGGCCCCGAGTTACT |
| ANXA1 | NM_000700 | AGCCTTTAAATCATTTTTATATTATAACTCTGTATAATAGAGATA  CAGCTACATAGACATCTTTCTCATGTATTGT  CCCAAACCATAAAACC |
| ARMET | NM_006010 | AGATCTGTGAGAAGCTTAAGAAGAAGGA  GAGCTTCTTCAGGTCCACTGT  TCGATCTGCTTGTCATACTT |
| CDH3 | NM_001793 | CCGTCCTGCATTTCTGGTTTC  ACGCAGAGATCCATCCGAATG  CAGACCCCAATGCCTC |
| CEACAM6 | NM_002483 | GGTCTCTCTTTGGCTGGAATTACAA  GGGCTTTTATCCTTCATGGGTTATG  TCCTGATGACACATTTC |
| EGFR | NM_201282 | CAGCAGTCCTTTGTAAACAGTGTTTT  GTGACTGAACATAACTGTAGGCTGAA  CACCCCATCCAATTTA |
| GAPDH | NM_002046 | ACCACTTTGTCAAGCTCATTTCCT  GAGGTCCACCACCCTGTTG  CTGTAGCCAAATTCG |
| GPRC5A | NM_003979 | GCACCCCAACCTCTGTAAATAGATT  CATTAGGAGACCATGCCCACTT  ACGGCTGCATTCTGT |
| IGFBP3 | NM_000598 | GACATTCTGCCTACCTATTAGCTTTTCTTTA  GGTCAATAACAAAGGGAAAGATATTTTTTTAATGGTAAA  CATTGCAAGACAAACTT |
| IL8 | NM_000584 | GGTAGTGCTGTGTTGAATTACGGAATA  GTACATAATAAACAAGTTTCAACCAGCAAGA  ACAGCCAAAACTCC |
|  |  |  |
|  |  |  |
| **Gene** | **Accession Number** | **Primers** |
| JAG1 | NM_000214 | TGTCCAGTAAGATCACTGTTTAGATTTGC  TGAACTTCGTAATAGCACTTTGATTTCCT  ACTGCCTGCCTTAAGTG |
| KLK3 | NM_001648 | GCGGCGGTGTTCTGG  GTCCACACACTGAAGTTTCTTTGG  CAGCTGCCCACTGCAT |
| KRT7 | NM_005556 | GGAGTGGGAGCCGTGAAT  CCACCGCCACTGCTACTG  ATCTCTGTGATGAATTCC |
| LCN2 | NM_005564 | CAGTGTATCGACGGCTGAGT  CCAGCTCCCTCAATGGTGTTC  CTGGCGGCACCTGTG |
| MMP11 | NM_005940 | GCCCTGGCTGAGCAACT  ATGCAGGCACCTACCAAGAC  CCTGCCCTACAGCCC |
| MTA1 | NM_004689 | GGCTGGAGCGGAGATGAG  CGGCCTCCGGGCAAA  CCACCCCGTGCCCC |
| NME1 | NM_000269 | CCGTGGAGACTTCTGCATACAAG  CTGCACTCTCCACAGAATCAC  TTGGCAGGAACATTATAC |
| NMU | NM_006681 | GAAGAAGGTCAGCAGGGTTCATT  CATTTTGTATTCCATAGCATTGCTCTGT  TAGCTGGCATCCATTTTA |
| NNMT | NM_006169 | CCTCGGCAGCCTACTGAAG  CTGCTCACCAATCATGTAGTAGCT  CTTGAGCGCATCCATG |
| NQO1 | NM_000903 | TGACTGGCACTGGTGGTTTT  GCAGTGAAGATGAAGGCAACAAAAT  CAGCCGTCAGCTATTG |
|  |  |  |
|  |  |  |
|  |  |  |
| **Gene** | **Accession Number** | **Primers** |
| PLAT | NM_000930 | AGTAGCATGAGAGAATTGTATCATTTGAACA  GGGTTGTGGCAACAGAAAGTAAAA  CTAGGCTTCAGCATATTT |
| POSTN | NM_006475 | CAAATTGAGTAATTCAGAAAAACTCAAGATTTAAGTTAAAAA  CTTGTTACAGTAAAAGAGGTATAAAGTCCTGTT  CCCAAGTCCAAACCAC |
| PRSS2 | NM_002770 | CTCGAGGGAGGCAAGGATTC  TCCTTGGAGCTCTCCATTGGA  CTGCCAGGGTGATTCT |
| PSCA | NM_005672 | ACGTGAGTTCCTGGGAGTCT  GGAGCCCCACGAATGTGA  CCAGGCCCCATCTCT |
| PSMB6 | NM_002798 | CAAGGAAGAGTGTCTGCAATTCAC  CTGCCAGGCGGATCACT  CCCGCTCCATGGCCAA |
| S100P | NM_005980 | GTGCTGATGGAGAAGGAGCTA  TCCACGGCATCCTTGTCTTTT  CCACTCTGCAGGAAGC |
| SLPI | NM_003064 | TGTGTGGGAAATCCTGCGTTT  GACTCCAGAGCCTCCTCCATAT  CCCTGTGAAAGCTTGATTC |
| SPINK1 | NM_003122 | TCCCAATGAATGCGTGTTATGTTTTG  TTCTCAGCAAGGCCCAGATTTT  CTGGCGTTTCCGATTTT |
| TFF2 | NM_005423 | GGTCCCCTGGTGTTTCCA  ACCTCCATGACGCACTGATC  CCCCTCCCAAAGCAA |
| TIMP1 | NM_003254 | GGCTTCACCAAGACCTACACT  GTCCGTCCACAAGCAATGAG  ATGCACAGTGTTTCCC |
